# Supplementary material for: N460S in PB2 and I163T in nucleoprotein synergistically enhance the viral replication and pathogenicity of influenza B virus
Source: PLoS Pathog. 2025 Sep 8;21(9):e1013463. doi: 10.1371/journal.ppat.1013463 (PMC12431660; doi:10.1371/journal.ppat.1013463)
Supplement: S1 Table — (DOCX) [file ppat.1013463.s001.docx]

### S1 Table**.** Primers used to quantify mRNA expression of IBV genes and mouse-specific proinflammatory markers

| Gene | Forward Primer (5′-3′) | Reverse Primer (5′-3′) |
| --- | --- | --- |
| For influenza B virus | | |
| PB2 | GGGAAGTCATAATGGGAGCTAAT | GCACCAGTTCTTTGGTTGTTC |
| NP | GCTCGTAGTATGGTCGTTGTTAG | GGCTTCATACCCAACCATAGAG |
| hGAPDH | TCAAGGCTGAGAACGGGAAG | CGCCCCACTTGATTTTGGAG |
| For mouse-specific proinflammatory markers | | |
| β-Actin | GGTGGGAATGGGTCAGAAGGA | TGGCTGGGGTGTTGAAGGTC |
| TNF-α | GACAAGGCTGCCCCGACTACG | CTTGGGGCAGGGGCTCTTGAC |
| IL-1β | AAATCTCGCAGC AGCACATCAA | CCACGGGAAAGACACAGGTAGC |
| IL-6 | AGTTGCCTTCTTGGGACTGA | CCTCC GACTTGTGAAGTGGT |
| IL-8 | CAGCTGCCTTAACCCCATCA | CTTGAGAAGTCCATGGCGAAA |
| IFN-α | ACTCATTCTGCACTGGCCTCCA | ACTTCTGCTCTGACCACCTCCC |
| CCL5 | AGATCTCTGCAGCTGCCCTCA | GGAGCACTTGCTGCTGGTGTAG |
| TNF | GAACTGGCAGAAGAGGCACT | AGGGTCTGGGCCATAGAACT |
| IL-10 | GGTTGCCAAGCCTTATCGGA | ACCTGCTCCACTGCCTTGCT |
