# Supplementary figures and images for: N460S in PB2 and I163T in nucleoprotein synergistically enhance the viral replication and pathogenicity of influenza B virus
Source: PLoS Pathog. 2025 Sep 8;21(9):e1013463. doi: 10.1371/journal.ppat.1013463 (PMC12431660; doi:10.1371/journal.ppat.1013463)

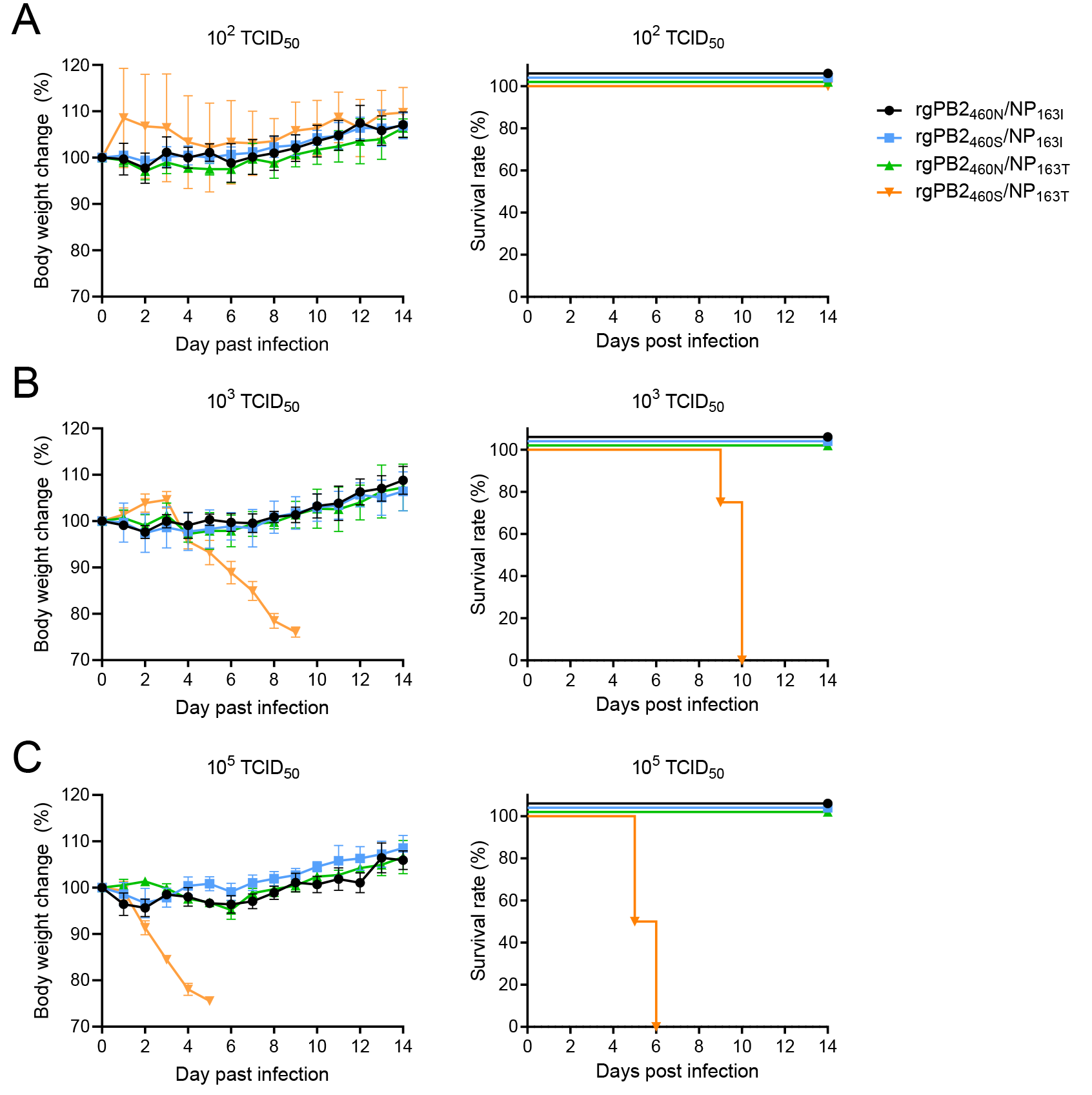

Supplement: S1 Fig — Body weight changes (left panels) and survival rates ((right panels) of six-week-old female BALB/c mice intranasally infected with 102 (A), 103 (B), 105 (C)TCID50 of indicated recombinant IBVs. (TIF) [file ppat.1013463.s004.tif]

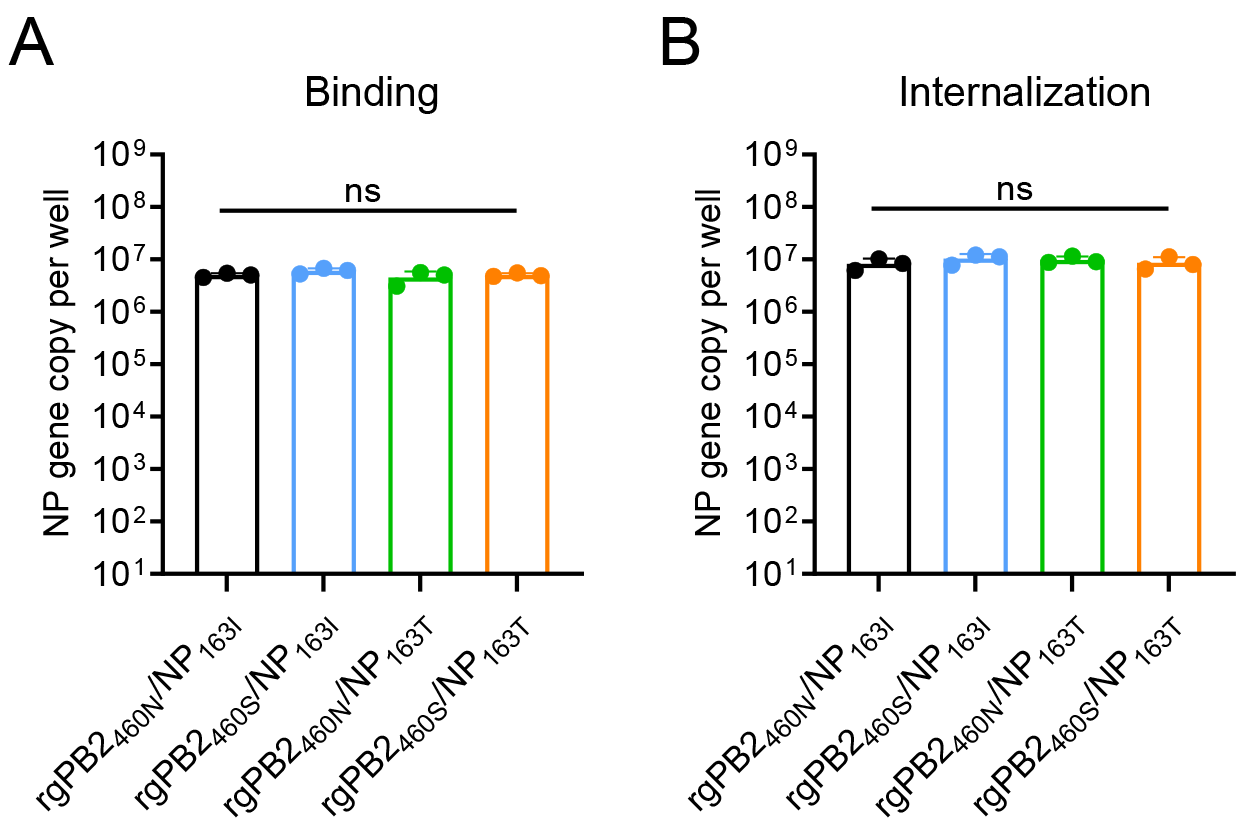

Supplement: S2 Fig — (A) NP RNA of viruses attached to the surface of MDCK cells. MDCK cells were incubated with each recombinant virus at 4 °C for 1 h to permit attachment, washed with ice‐cold PBS (pH 7.2) to remove unbound virus, and cell‐attached NP RNA was quantified by RT‐qPCR. (B) NP RNA of viruses internalized into MDCK cells. MDCK cells were incubated with each recombinant virus at 4 °C for 1 h, transferred to 33 °C for 1 h to allow internalization, then washed with acidic PBS (pH 1.5) to remove remaining surface‐attached virus. Intracellular NP RNA was measured by RT‐qPCR. ns, not significant. (TIF) [file ppat.1013463.s005.tif]

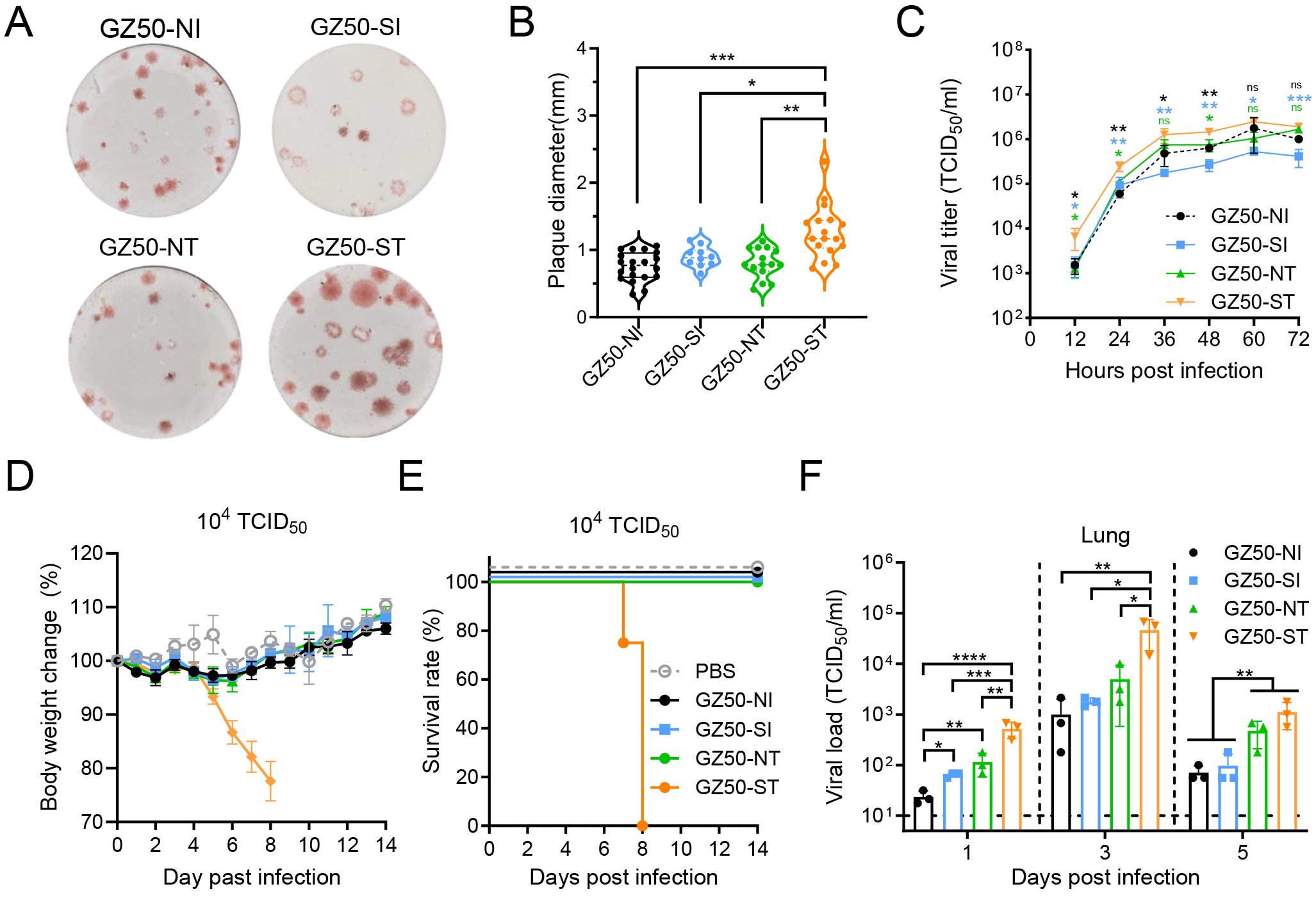

Supplement: S3 Fig — Reverse mutations (PB2-S460N and NP-T163I) were introduced into a more recently circulating IBV strain, B/Guangzhou/50/2022 B/Guangzhou/50/2022 (GZ50; Victoria lineage, clade V1A.3a.1; GISAID isolate EPI_ISL_19888228) by using reverse genetics. Four viruses were generated on the GZ50 backbone: GZ50-NI (PB2-460N/NP-163I; double-reverse mutant), GZ50-SI (PB2-460S/NP-163I; single-substitution mutant), GZ50-NT (PB2-460N/NP-163T; single-substitution mutant), GZ50-ST (parental PB2-460S/NP-163T). (A) Representative plaque assays of these four GZ50-derived viruses on MDCK cells, stained 72 h post-infection. (B) Plaque diameters for each recombinant IBV were determined by Adobe Photoshop (CC 2019). (C) Growth kinetics of the indicated recombinant viruses in MDCK cells at an MOI of 0.01. Statistical significance is indicated using asterisks in different colors for clarity: black, blue, and green asterisks denote significant differences of GZ50-NI vs. GZ50-ST, GZ50-SI vs. GZ50-ST, and GZ50-NT vs. GZ50-ST, respectively. Body weight changes (D) and survival rates (E) of six-week-old female BALB/c mice (n = 4 per group) intranasally infected with 104 TCID50 of each GZ50-derived virus or PBS. (F) Lung viral titers measured at 1, 3, and 5 dpi (n = 3 per time point) following infection with 104 TCID50 of the indicated viruses. The limit of detection is indicated by the horizontal dashed line. * p < 0.05; **, p < 0.01; ***, p < 0.001; ****, p < 0.0001; ns, not significant. (TIF) [file ppat.1013463.s006.tif]

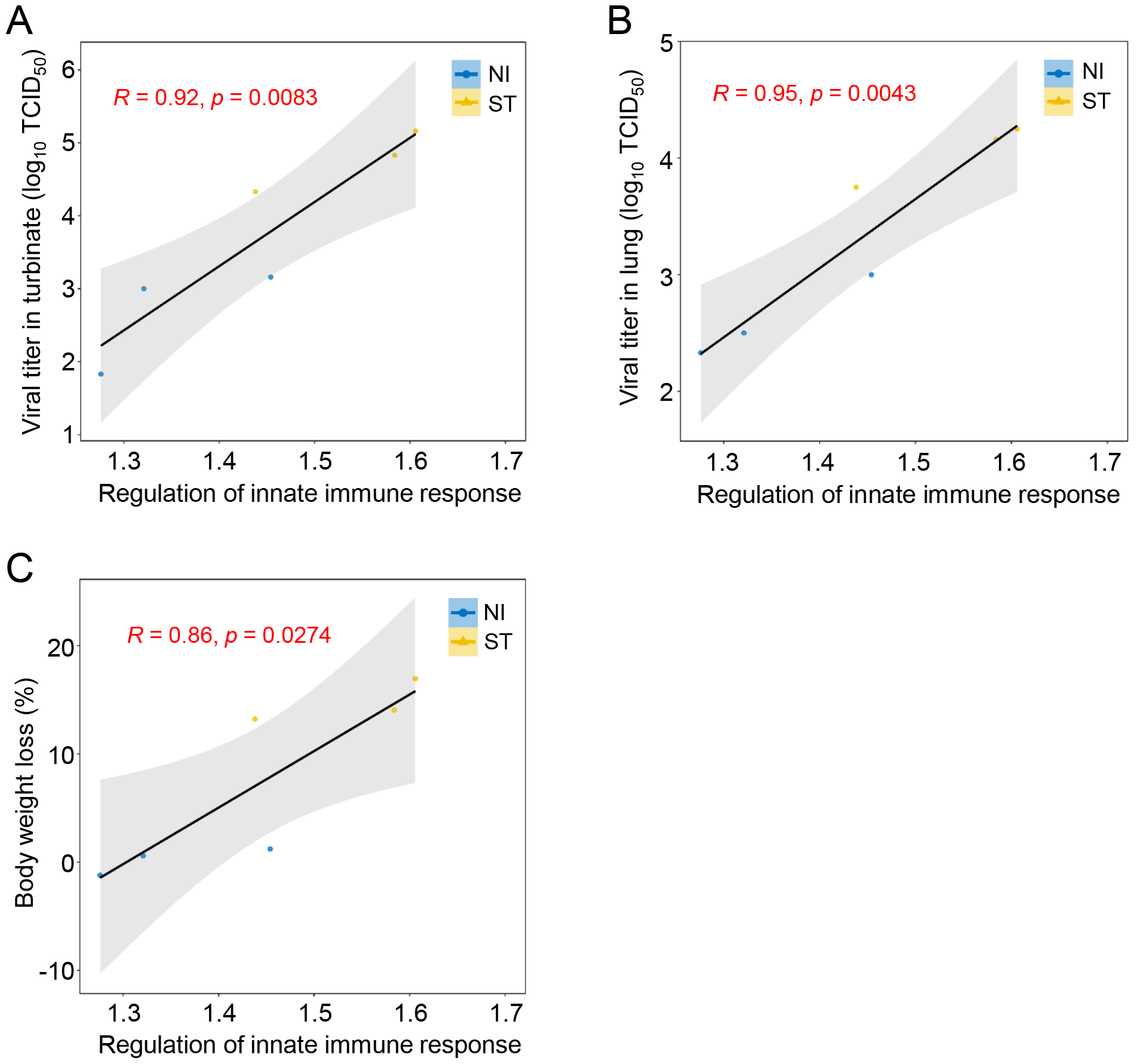

Supplement: S4 Fig — Single-sample gene set enrichment analysis (ssGSEA) enrichment scores for the “Regulation of innate immune response” pathway were calculated from lung transcriptomes of mice infected with rgPB2460N/NP163I (NI) or rgPB2460S/NP163T (ST) viruses at 5 dpi. Panels show the correlation between ssGSEA scores and body-weight loss (A), viral titer in nasal turbinate (B), and viral titer in lung tissue at the same timepoint (C). Regression lines (solid) and 95% confidence intervals (shaded) are displayed. Pearson’s R and p values are indicated in red. (TIF) [file ppat.1013463.s007.tif]
