# Supplementary material for: N460S in PB2 and I163T in nucleoprotein synergistically enhance the viral replication and pathogenicity of influenza B virus
Source: PLoS Pathog. 2025 Sep 8;21(9):e1013463. doi: 10.1371/journal.ppat.1013463 (PMC12431660; doi:10.1371/journal.ppat.1013463)
Supplement: Attachment — Submitted filename: Response to Reviewers.docx [file ppat.1013463.s009.docx]

**Response letter**

Additional Editor Comments:

PLOS and PLOS Pathogens have a specific policy regarding research that would fall into the category of “dual use research of concern” (https://journals.plos.org/plospathogens/s/ethical-publishing-practice#loc-biosecurity-and-dual-use-research-of-concern). Your submission was referred to the PLOS Dual Use Committee for an assessment of the potential risks versus benefits of publication. The Committee determined that there are some potential risks of publishing your submission, but these may be mitigated if the authors can address the following concerns. Please address these as part of your revisions, and include your responses in your Response to Reviewers when you resubmit. Your responses may be referred back to the DURC Committee to confirm that the risks have been adequately mitigated, so please ensure your revisions are thorough.

1.	The authors should include details about biosafety and containment measures as well as institutional and regulatory oversight of the work conducted.

**Response:** We appreciate the Committee’s review and the editor’s suggestion. We have updated the Ethics statement (lines 398-400) and Cells and viruses section (lines 408–412) to include detailed biosafety and oversight information:

Virus work: All virus isolation, rescue, propagation, and in vitro assays with authentic influenza B virus were conducted under enhanced Biosafety Level 2+ (BSL-2+) conditions at the First Affiliated Hospital of Guangzhou Medical University, in compliance with the People’s Republic of China Biosafety Law and GB 19489-2008 “Biosafety Requirements for Pathogenic Microorganism Laboratories.”

Animal studies: Animal challenge experiments were approved by the Institutional Animal Care and Use Committee of Guangzhou Medical University (Permit No. 20220605) and performed in an Animal Biosafety Level 2 (ABSL-2) facility at our institution.

We believe these additions demonstrate our full compliance with all relevant biosafety regulations.

2.	The authors should include some more information as to the rationale for this study, and provide additional details of the clinical context that may be relevant to the evaluation of potential biosecurity concerns.

**Response:** We appreciate the suggestion to include additional rationale and clinical context to address potential biosecurity concerns. We have responded to points a–d and incorporated this information into the main text (lines 123-126). Furthermore, An amino acid variant frequency analysis of all available IBV genomes in GISAID demonstrates that the N460S and I163T substitutions are prevalent in historical strains. Our reverse-genetics viruses therefore do not introduce any new gain-of-function beyond what is already present in circulating strains.

a. Why were these isolates specifically chosen for further experimentation?

**Response:** Following initial virus recovery of GZ0215 in MDCK cells, we performed routine plaque purification and observed two distinct plaque phenotypes. From these, we isolated GZ0215-01 and GZ0215-06, which—despite deriving from the same clinical sample—consistently yielded plaques that differed in size by ~4-fold. This clear phenotypic heterogeneity prompted their selection for detailed genetic and functional analysis.

b. Were there specific clinical symptoms from the patient that prompted further investigation?

**Response:** The patient—a 12-year-old outpatient—presented with an uncomplicated upper respiratory infection (fever, nasal congestion, rhinorrhea, sneezing) and received only supportive care. No atypical symptoms or treatments prompted the study; rather, the plaque-size disparity in vitro drove further characterization.

c. Was the initial culture of the isolates routine, and the increased plaque size of one of the isolates was the prompt for further investigation?

**Response:** Yes, initial virus isolation was performed on MDCK cells as per standard protocol. Yes, the observed difference in plaque size prompted further characterization.

d. What was the sequence of experiments for this study?

**Response:** Following initial virus recovery in MDCK cells from the oropharyngeal specimen, we performed routine plaque purification and identified two distinct plaque phenotypes, which we designated GZ0215-01 and GZ0215-06. Meanwhile, we conducted whole-genome sequencing on the original viral stock as well as on both plaque isolates. Finally, using the GZ0215-01 background, we generated recombinant viruses by reverse genetics for subsequent functional analyses.

3.	The first line of the concluding statement could be misinterpreted as recommending the best way to enhance the pathogenicity of IBV. This sentence should be rephrased in a more passive voice to prevent any assumptions of DURC or misinterpretation.

**Response:** We appreciate this important suggestion. To avoid any implication of recommendation, we have rephrased the opening line of the concluding statement into passive voice. The revised text now reads:

“In summary, the PB2-N460S and NP-I163T substitutions have been shown to synergistically enhance polymerase activity, viral replication, pathogenicity, and host inflammatory responses in IBVs, providing insight into molecular determinants of IBV pathogenesis and informing future prevention and treatment strategies.”

Reviewers' Comments:

Reviewer #1:

This study identifies the critical roles of PB2-N460S and NP-I163T mutations in enhancing IBV replication and pathogenicity. The co-existence of these substitutions synergistically enhances polymerase activity, promotes viral replication, increases pathogenicity, and amplifies host inflammatory responses, potentially contributing to severe IBV infections. While demonstrating novelty, the manuscript requires substantial improvements in result interpretation, logical coherence, and experimental validation. Key concerns include insufficient discussion depth, ambiguous conclusions, and methodological uncertainties that necessitate comprehensive revisions.

Major issues:

1. paragraph 77-78: The statement regarding NP protein distinctions between IAVS and IBVS requires expansion. Please provide explicit comparative analysis of their structural/functional differences rather than simply stating dissimilarity.

**Response:** We appreciate the reviewer’s insightful and thorough critique and suggestions, which has greatly strengthened our manuscript.

In the revised manuscript, we have expanded paragraph 83–94 to include detailed, side‑by‑side comparisons of IAV and IBV RNPs, as follows (lines 83–94):

“In both virus types, each RNA segment is packaged into a ribonucleoprotein (RNP) complex, in which the 5′ and 3′ terminal of viral RNA are bound by one polymerase heterotrimer while the RNA is covered by multiple NP monomers [14]. Despite these similarities, IAV and IBV RNPs differ markedly in sequence, structure, and function [15]. Cryo EM tomography shows that IAV RNPs adopt a tightly ordered “7 + 1” oval arrangement [16], whereas IBV RNPs appear as loosely packed, variably oriented bundles without a defined “7 + 1” pattern [17]. At the polymerase level, IAV and IBV polymerase heterotrimers engage host RNA polymerase II and ANP32 family cofactors via distinct interfaces and suggesting distinct RNA synthesis and RNP assembly mechanisms [18, 19]. Sequence differences in the PB2 cap binding domain also give IBV PB2 an inverted substrate recognition mode and reduced m⁷GDP affinity compared to IAV PB2 [20]. Finally, IBV NP carries an a unique N terminal extension of approximately 50–70 residues—essential for viral viability, nuclear import, and efficient transcription and replication—that is absent in IAV NP [21, 22].”

2. paragraph 115-117: Why do GZ0215-01 and GZ0215-06 have different pathogenicity in mice with the same infection dose? If GZ0215-01 has low replication ability, the same infection dose should contain more virus particles. Is it because of the difference in the ability of MDCK cells and mouse cells to replicate the virus? The authors should explore this issue and provide their TCID50 detection method and results in the supporting documents.

**Response:** Thank you for this insightful question. We used the WHO-recommended ELISA-based TCID_50_ assay on MDCK cells to determine all viral titers, and have added these details to the Methods (lines 455–462). It is indeed true that, because GZ0215-01 replicates less efficiently, a dose normalized to the same TCID_50_ as GZ0215-06 may contain a higher number of physical virions. Nevertheless, GZ0215-01 still produced markedly lower pathogenicity in mice, reinforcing our conclusion that intrinsic replication capacity—rather than particle count—is the primary driver of virulence.

3. paragraph 149-154: Potential adaptive mutations during in vivo passage must be systematically excluded. Include sequencing data from post-infection isolates to confirm phenotypic stability.

**Response:** We sequenced viral genomes from both MDCK cell supernatants (72 hpi) and mouse lung tissues (5 dpi) for all four recombinant viruses (Supplemental Tables 2 and 3). No adaptive mutations were detected at PB2-460 or NP-163 in any sample. In MDCK cells, we observed only low-frequency (<15%) host-adaptive substitutions elsewhere in the genome. In mouse lungs, no high-frequency (>50%) host-adaptive mutations were identified. We did detect a PB2-527 polymorphism at moderate frequency (25–49%) across all viruses; however, its frequency in the PB2-460S/NP-163T double mutant was lower than in the other variants. These findings indicate that host-adaptive substitutions did not contribute to the enhanced replication and pathogenicity of the PB2-460S/NP-163T double mutant. These data are now included in Supplemental Tables 2 and 3 and have been incorporated into the Results section (Lines 154–158, 194-200) and Discussion section (Lines 350-360).

4. paragraph 169-170:The causality between enhanced inflammation and mutations requires rigorous validation. The observed immune responses may represent secondary effects of increased viral load rather than mutation-specific immunomodulation. Revise conclusions accordingly and address this confounding factor in transcriptome analysis.

**Response:** We agree that higher innate immune response may largely reflect increased viral load rather than a direct immunomodulatory effect of the amino acid substitutions. To address this, we performed single-sample gene set enrichment analysis (ssGSEA) on our lung transcriptome data to identify innate immune responses correlated with both body weight loss and airway viral titers. We found that enrichment scores for the “Regulation of innate immune response” pathway strongly correlated with nasal turbinate titer (R = 0.92, p = 0.0083), lung titer (R = 0.95, p = 0.0043), and body-weight loss (R = 0.86, p = 0.0274) at 5 dpi (Supplemental Figure 4). These results indicate that the enhanced innate immune responses in rgPB2460S/NP163T infected mice are closely associated with increased viral replication and pathogenicity. Accordingly, we have updated the Results (lines 241–248) and section conclusion (line 252) to include these correlations and added the detailed methodology under “Immune Pathway Enrichment and Correlation Analysis” in the Supplemental Methods.

5.paragraph 206-207: The mRNA upregulation level is greater than the protein expression upregulation level. Does this indicate that 163T is at a disadvantage in the expression stage?

**Response:** Thank you for pointing this out. It’s common for large mRNA changes not to translate into equivalent protein increases, due both to post-transcriptional regulation (e.g., translation rate) and the narrower linear range of Western blot detection. In our case, although NP-163T mRNA rises ~30-fold, protein levels increase only ~3-fold; we do not interpret this as a “disadvantage” of the 163T variant.

6. paragraph 229-233: Please run interface analysis and affinity analysis to quantify whether and how the characterization is enhanced.

**Response:** Thank you for your insightful suggestion. We have now added quantitative interface and affinity analyses to paragraph 287–292:

“PRODIGY calculations then showed that the PB2-N460S substitution yielded a more favorable binding free energy (ΔG = –7.1 vs. –6.5 kcal/mol) and a lower dissociation constant (Kd = 6.3 × 10-6 M vs. 1.6 × 10-5 M at 25 °C) (Figure 6B and C), indicating tighter PB2–NP binding. We speculate this stabilization arises from reduced steric hindrance due to the shortened side chain after the N-to-S substitution, enabling closer proximity between PB2 and NP-4 in the 460 region.”

The corresponding panels have been added to Figure 6B and 6C, and the Methods (“Structural Prediction and Interaction Analysis”) have been updated to describe these PRODIGY and PDBePISA analyses.

7. paragraph 241: The author repeatedly mentioned the pathogenic mechanism, but the main cause of the pathogenicity mentioned in the article is the enhanced viral replication, which is the reason for the degree of pathogenicity, not its pathogenic mechanism. Body weight changes were also found in mice challenged with the original strain.

**Response:** We appreciate this insightful comment. Indeed, in our study, we identified the PB2‑N460S/NP‑I163T dual substitutions as key molecular determinants of IBV pathogenicity, acting by enhancing viral replication and amplifying inflammation. To clarify, we have revised lines 300-301 to: “Unlike IAVs, whose pathogenesis is well‑characterized, the molecular determinants of IBV pathogenicity remain poorly understood.” We have also replaced all instances of “pathogenic mechanism” with “pathogenesis” or “determinants of pathogenicity” throughout the manuscript.

Additionally, although GZ0215‑01 infected mice experienced minor weight loss at the highest dose, they exhibited substantially lower pathogenicity than mice infected with GZ0215‑06.

8. paragraph 254: Please cite references

**Response:** We have added appropriate citations (references 38 and 44).

9. paragraph 282-287: Regarding PB2-460S and NP-163T, the prevalence should not only be mentioned in the discussion, but should be more intuitively shown in the results, especially the currently prevalent V1A.3a.2 paragraphage viruses.

**Response:** We have done prevalence analysis of PB2-460 and NP-163 across all available IBV genomes from GISAID (Figure α). This revealed that the PB2-460N and NP-163I substitutions occurs at <4% and <0.2% annually, respectively, whereas the PB2-460S/NP-163T combination is found in >99% of sequences. We have incorporated these data into the Discussion (lines 341-348) to highlight the near‐fixation of S/T in circulating IBVs.

The fact that we isolated the rare N/I variant directly from the patient sample likely reflects genuine intrahost viral heterogeneity. Our passage-1 whole‐genome sequencing on MDCK cells confirmed these substitutions at low frequency, consistent with their lower replication fitness—explaining why they are underrepresented in global prevalence data.

By contrasting this natural “loss‐of‐function” N/I clone with the dominant S/T genotype, we provide a biologically relevant control that strengthens our mechanistic conclusions and underscores the applicability of our findings for surveillance and vaccine design.

**Figure α. Annual prevalence of PB2-460 and NP-163 variants in global IBV genomes**

10.paragraph 304-309: In addition to the current prevalent situation, the authors should also focus on whether GZ0215-06 can be used to replace the high-yield vaccine backbone or whether GZ0215-01 can be used in the design of attenuated vaccines.

**Response:** Thank you for this insightful question regarding vaccine development. As noted, PB2-N460S and NP-I163T are highly prevalent among circulating IBV isolates and are already incorporated into current high-yield vaccine backbones. In contrast, GZ0215-01 retains the ancestral PB2-460N/NP-163I residues associated with reduced virulence, which could inform the design of live-attenuated vaccines.

Reviewer #2:

The findings highlight the critical role of the PB2-N460S and NP-I163T substitutions in enhancing the replication and pathogenicity of IBVs. These substitutions, when present together, synergistically enhance polymerase activity, promote viral replication, increase pathogenicity, and elevate the host inflammatory response, potentially contributing to the severity of IBV infections. These findings provide new insights into the molecular determinants of IBV pathogenesis, highlighting the synergistic effect of PB2-N460S and NP-I163T in enhancing viral fitness and worsening disease outcomes. However, there are several areas that require further clarification and improvement.

Major issues:

1.	The authors mention in lines 253-254 of the Discussion that amino acid variations in PB2 and NP may affect viral replication and host tropism. While this study primarily demonstrates that the combined mutations enhance viral replication and pathogenicity in cellular and mouse models, could these mutations also alter cross-species infectivity?

**Response:** We appreciate this important point. Unlike IAV, IBV has a narrow, primarily human host range. Our findings in MDCK cells—a highly permissive influenza cell line—and in mice—a common mammalian model—demonstrate enhanced fitness in these systems, but do not imply that PB2-N460S/NP-I163T alter cross-species infectivity.

2.	Line 277: There is a missing space between "between" and "PB2-460"

**Response:** Thank you for catching this. This has been corrected.

3.	Figure 2C shows that the combined mutant strain exhibits higher viral load. Could you provide the viral entry load at 2 hours post-infection? This would help determine whether there are differences in viral entry efficiency into cells.

**Response:** We performed a viral entry assay in MDCK cells and found that all four recombinant viruses exhibited comparable binding and entry efficiencies, ruling out entry differences as a factor in their replication phenotypes. These data have been added to Supplemental Figure 2 and to the Results section (lines 152–154), and the corresponding method has been added to Supplemental Methods section.

4.	For Figure 5D, statistical analysis is missing for the right panel.

**Response:** Thank you for pointing this out. We have now added the missing statistical analysis to the right panel of Figure 5D. This subfigure has also been double-checked with an extra independent experiments, as suggested in question #7, to ensure the robustness of the results.

5.	The research showed that the PB2 - N460S/NP - I163T dual substitutions led to enhanced immune activation and excessive inflammation. How do you think this hyper - inflammatory state relates to the development of drug resistance in influenza B virus? Are there any potential implications for antiviral therapy?

**Response:** We appreciate this important question. Although our study did not directly assess antiviral susceptibility, we can offer the following considerations. Our data showed that PB2-N460S/NP-I163T dual substitutions increased viral replication, which in turn drove the hyper-inflammatory response observed in infected lungs. Because viral replication itself is a key trigger of inflammation, initiating antiviral therapy at the earliest possible time (e.g., oseltamivir or baloxavir) could both limit viral burden and blunt downstream cytokine storms. In support of this, our previous work published in MED (doi: [10.1016/j.medj.2023.11.001](https://doi.org/10.1016/j.medj.2023.11.001) ) demonstrated that delayed antiviral treatment often fails to clear high‐titer virus effectively, and even when viral loads fall, the established hyper-inflammation persists, leading to severe lung injury and multi-organ failure.

6.	Does the dual mutation lead to a higher rate of severe cases clinically compared to the control group?

**Response:** We appreciate this critical question. In our mouse model, the PB2-N460S/NP-I163T dual substitutions significantly enhanced viral replication and pathogenicity, demonstrating that these combined mutations are key drivers of increased virulence. However, because the S/T genotype is nearly fixed in annual circulating IBVs, direct comparisons of clinical severity between genotypes are not feasible.

7.	In Figure 5D, the expression of 163I-NP appears notably low despite high loading control levels. Since the authors used plasmid transfection for protein expression, could this result from low transfection efficiency? Including an HA-tagged plasmid as a transfection efficiency control would help clarify this.

**Response:** Thank you for this valuable suggestion. We have repeated the Figure 5D experiments in three additional independent biological replicates (Figure βA). To control for transfection efficiency and antibody recognition, we employed a pVITRO2-MCS dual-expression plasmid (InvivoGen, Cat. No. pvitro2-nmcs), in which HA-tagged NP (163I or 163T) is driven by the hFerL promoter and GFP by the FerH promoter (Figure βB). Equivalent GFP levels confirmed similar transfection efficiencies for both NP variants, and detection with an anti-HA antibody ruled out any difference in antibody recognition due to the I163T substitution. The results still showed that NP-163T exhibited higher protein expression than NP-163I, indicating that the I163T substitution enhances NP protein accumulation (Figure βC).

**Figure β. I163T substitution enhances NP expression (related to Figure 5D)**

(A) HEK293T cells were transfected with pCAGGS expression plasmids encoding NP-163I, NP-163T, or empty vector. At 24 h post-transfection, cell lysates were analyzed by Western blot using rabbit anti-NP antibody. GAPDH serves as a loading control.

(B) Schematic of the pVITRO2-GFP/NP plasmid. The pVITRO2-MCS dual-expression plasmid carries a GFP gene under the control of the hFerL promoter and an HA-tagged NP coding sequence (either NP-163I or NP-163T) under the control of the FerH promoter.

(C) HEK293T cells were transfected with pVITRO2-MCS dual-expression plasmids encoding GFP alone (pVITRO2-GFP) or dual-expressing GFP and HA-tagged NP genes (163I or 163T). Cell lysates were detected with anti-HA antibody to detect NP variants, anti-GAPDH antibody for loading control, and anti-GFP antibody to confirm equivalent transfection across samples.

8.	Figures 3D and 3E show that the combined mutations result in higher viral loads in nasal turbinates and lungs. Could the author further analyze the differences in viral loads between upper and lower respiratory tracts? Would such differences correlate with disease severity?

**Response:** Thank you for pointing this out. Figures 3D and 3E were intended to compare viral loads between virus variants within each tissue (nasal turbinate or lung), not to contrast absolute titers across these anatomically and structurally distinct sites. Because the turbinate and lung differ markedly in mass, cellular composition, and susceptibility, direct cross‐tissue comparisons of viral titer may not be appropriate.

Reviewer #3:

The authors identified two amino acid substitutions—N460S in PB2 and I163T in NP—from IBV clinical isolates with distinct replication and pathogenicity phenotypes. Using reverse genetics, they demonstrated that while neither mutation alone significantly impacted viral replication or pathogenicity, their combination synergistically enhanced both traits. These findings offer valuable insights into the functional interplay between PB2 and NP, key components of the viral RNP complex. However, the approach lacks novelty, and the extent to which the results can be generalized to other IBV strains remains unclear, limiting the overall impact of the study.

**Response:** We appreciate your recognition of the importance of our findings and your suggestions.

Major issues:

1. It would greatly enhance the manuscript if the authors evaluated the stability of the reverse-genetic viruses after 72 hours of replication in MDCK cells. Specifically, the emergence of any additional mutations should be investigated. Likewise, it would be valuable to determine whether further mutations arise after 5 days post-infection (dpi) in mice.

**Response:** We sequenced viral genomes from both MDCK cell supernatants (72 hpi) and mouse lung tissues (5 dpi) for all four recombinant viruses (Supplemental Tables 2 and 3). No adaptive mutations were detected at PB2-460 or NP-163 in any sample. In MDCK cells, we observed only low-frequency (<15%) host-adaptive substitutions elsewhere in the genome. In mouse lungs, no high-frequency (>50%) host-adaptive mutations were identified. We did detect a PB2-527 polymorphism at moderate frequency (25–49%) across all viruses; however, its frequency in the PB2-460S/NP-163T double mutant was lower than in the other variants. These findings indicate that host-adaptive substitutions did not contribute to the enhanced replication and pathogenicity of the PB2-460S/NP-163T double mutant. These data are now included in Supplemental Tables 2 and 3 and have been incorporated into the Results section (Lines 154–158, 194-200) and Discussion section (Lines 350-360).

2. The authors have not assessed the frequency of the PB2-N460S and NP-I163T mutations in circulating seasonal influenza B virus (IBV) strains.

**Response:** We have done prevalence analysis of PB2-460 and NP-163 across all available IBV genomes from GISAID (Figure α). This revealed that the PB2-460N and NP-163I substitutions occurs at <4% and <0.2% annually, respectively, whereas the PB2-460S/NP-163T combination is found in >99% of sequences. We have incorporated these data into the Discussion (lines 341–348) to highlight the near‐fixation of S/T in circulating IBVs.

The fact that we isolated the rare N/I variant directly from the patient sample likely reflects genuine intrahost viral heterogeneity. Our passage-1 whole‐genome sequencing on MDCK cells confirmed these substitutions at low frequency, consistent with their lower replication fitness—explaining why they are underrepresented in global prevalence data.

**Figure α. Annual prevalence of PB2-460 and NP-163 variants in global IBV genomes**

3. It would be interesting to investigate whether the PB2-N460S and NP-I163T mutations synergistically enhance viral replication and pathogenicity when present in different influenza B virus backgrounds.

**Response:** We appreciate this insightful suggestion. To test whether the synergistic effect of PB2-N460S/NP-I163T extends beyond GZ0215, we introduced the reverse mutations (PB2-S460N and NP-T163I) into a more recently circulating IBV strain, B/Guangzhou/50/2022 (Victoria lineage, clade V1A.3a.1; GISAID isolate EPI_ISL_19888228), which we confirmed is lethal in mice. Using reverse genetics, we rescued the parental GZ50 (PB2-460S/NP-163T; “GZ50-ST”), the single-substitution mutants GZ50-NT (PB2-460N/NP-163T) and GZ50-SI (PB2-460S/NP-163I), and the double-reverse mutant GZ50-NI (PB2-460N/NP-163I). In MDCK cells, GZ50-NI, GZ50-SI, and GZ50-NT produced indistinguishable plaque sizes and comparable replication kinetics, whereas GZ50-ST generated significantly larger plaques and higher titers (Supplemental Figure 2A–C). In BALB/c mice challenged with 10^4^ TCID_50_ of each virus, mice infected with GZ50-NI, GZ50-SI, or GZ50-NT exhibited mild to no body-weight loss, but all GZ50-ST–infected mice lost > 25% body weight and were humanely euthanized (Supplemental Figure 2D–E). Lung titers at 1, 3, and 5 dpi were highest in GZ50-ST–infected mice compared to the three mutants (Supplemental Figure 2F). These results indicate that, in the GZ50 background, restoring PB2-460N/NP-163I attenuates replication and pathogenicity, mirroring our GZ0215 findings and confirming that the PB2-N460S/NP-I163T synergy is conserved across diverse IBV strains. We have added these data to Supplemental Figure 2, updated the Results (lines 202–216), and included a brief discussion (lines 362–368).

Reviewer #4:

In the study entitled “N460S in PB2 and I163T in nucleoprotein synergistically enhance the viral replication and pathogenicity of influenza B virus” by Gao et al., the authors assess molecular determinants of Influenza B virus (IBV) pathogenesis. To this end, genomic analysis of two influenza B virus (IBV) isolates, recovered from a patient’s single swab specimen, was performed and identified two non-synonymous substitutions – PB2-N460S and NP-I163T – as potential molecular drivers of phenotypic differences. Recombinant IB viruses carrying either or both mutations were generated and utilized in in vitro and in vivo approaches to demonstrate that individual mutations had no significant impact while their combination enhanced polymerase activity, replication efficiency, and pathogenicity, with severe disease outcome in the mice animal model. Next, Gao et al. study the effect of PB2-N460S and NP-I163T substitution on host immune response revealing an enhanced immune activation with elevated expression of antiviral and pro-inflammatory genes in transcriptomic profiles. Finally, using co-immunoprecipitation approaches and minigenome reporter assay, the authors demonstrate an enhanced PB2-NP binding and higher polymerase activity in the combinatorial presence of 460S and 163T. Structural modeling positioned PB2-N460S at the PB2-NP interface and NP-I163T at a distal site, suggesting indirect functional coupling.

This is an interesting study with valuable information on molecular determinants of Influenza B viruses. Although the study design is mostly sound, there are a few comments that the authors should consider to improve the quality and clarity of this manuscript.

**Response:** We appreciate the reviewer’s concise and thoughtful summary of our work and recognition of the novelty and value of our findings. We also appreciate your positive evaluation and constructive suggestions. In the sections that follow, we have addressed each of your comments in detail to improve the clarity, rigor, and presentation of our manuscript.

Minor Issues:

1. Please provide descriptions of the disease severity and disease outcome of the IBV patient from whom the isolate B/Guangzhou/0215/2012 (GZ0215) originated. Additionally, was the patient under treatment? This information is relevant for evaluating the clinical relevance since the manuscript discusses pathogenesis.

**Response:** The isolate was obtained from a 12-year-old boy who attended our outpatient clinic on February 16, 2012, with acute upper respiratory tract infection. He did not require hospitalization and presented with a high fever (39.3 °C), moderate nasal congestion, rhinorrhea, and frequent sneezing (severity score 3/3), but no cough, sputum production, chills, headache, or sore throat. He received only supportive care (fever reducers and hydration) and no antiviral or antibiotic therapy prior to sample collection. No further follow-up data are available.

We have added this description in the Results (lines 123–126).

2. Please provide more details on how patients were selected for inclusion or exclusion into the study to ensure the selection criteria was unbiased.

**Response:** Our study was designed to investigate IBV pathogenesis rather than conduct an epidemiological analysis; accordingly, we did not apply population‐level sampling methods. Instead, we used a consecutive‐sampling approach, including every available diagnostic leftover oropharyngeal swab from patients presenting with influenza‐like illness at the First Affiliated Hospital of Guangzhou Medical University in 2012.

Inclusion criteria: (1) RT-PCR–confirmed influenza B infection on admission; (2) ≥200 µL residual swab volume; (3) no antiviral therapy received prior to sampling.

Exclusion criteria: (1) co-detection of other respiratory viruses; (2) insufficient sample volume or poor RNA quality; (3) incomplete clinical metadata.

When these criteria were met, viral isolation was performed on MDCK cells, and isolates exhibiting relatively high cytopathic effect (CPE) were selected for plaque assays.

3. Ethic statement (line 313): “…retrospective nature of the residual sample collection”. Since amino acid substitutions may arise under selective pressure during antiviral therapy please clarify whether the sample used in this study was the initial diagnostic sample (diagnostic leftover) collected on admission to the hospital, or a follow-up sample.

**Response:** Thank you for this important point. The specimen analyzed was the initial diagnostic leftover collected on admission—prior to any antiviral therapy—not a follow-up sample.

4. Please clarify which material was used to study polymorphism – was the RNA retrieved directly from the oropharyngeal swab, or from the cell culture isolate prior to plaque purification? This distinction is important because if whole genome sequencing was performed on RNA derived from cell culture isolates rather than directly from the clinical sample, non-synonymous substitutions may have been introduced during virus propagation. That could influence the interpretation of polymorphism data and may not accurately reflect the viral population present in the patient.

**Response:** Thank you for highlighting this. The whole-genome sequencing of GZ0215 (Table 1) was performed on RNA extracted from cell-culture isolates after a single passage in MDCK cells (prior to plaque purification), rather than directly from the oropharyngeal swab. We understand that sequencing directly from clinical material would best reflect in vivo viral polymorphisms; however, the original swab specimen is no longer available. To minimize culture-adapted substitutions, we limited our analyses to this low-passage stock and observed no additional nonsynonymous mutations beyond those present in the initial isolate. Additionally, we sequenced viral genomes from MDCK cell supernatants for all four recombinant viruses (Supplemental Table 2), and no genetic polymorphism at the PB2-460 and NP-163 positions were observed across the four viruses, indicating that these genotypes were present in the original clinical sample and did not arise during viral isolation.

5. Ethics statement: Please indicate whether mice died during your mouse experiments or were euthanized as endpoint criteria were met at the respective time points. If endpoint criteria apply, please provide.

**Response:** Yes. Mice that lost ≥25% of their initial body weight were euthanized per our endpoint criteria. We’ve added this clarification to lines 511–512.

6. Please clarify which infection doses were used for GZ0215-01 and GZ0215-06 as there appears to be inconsistencies between the results section (line 114) and infection doses presented in Figure 1 panel C and D. Why were different infection doses used?

**Response:** Thank you for catching this. We have revised lines 132–134 to read: “BALB/c mice were intranasally challenged with tenfold serial dilutions of each strain: 10^2^–10^5^ TCID_50_ for GZ0215-01 and 10^1^–10^4^ TCID_50_ for GZ0215-06,” which now exactly matches Figure 1C and 1D. In pilot studies, GZ0215-01 exhibited lower pathogenicity and therefore required the higher dose range (10^2^–10^5^ TCID_50_) to attempt LD_50_ determination (which remained undetectable). By contrast, the more virulent GZ0215-06 yielded a measurable LD_50_ using the lower dose range (10^1^–10^4^ TCID_50_).

7. An interesting finding is the increased mRNA expression levels of the PB2-460N and NP-163T variants compared to the respective constructs without amino acid substitution. The coding regions of PB2 and NP were cloned into the pCAGGS vector under the control of an RNA polymerase II promoter, and expression experiments were performed in the absence of other viral components potentially affecting expression. Please explain how this enhanced mRNA level could be achieved despite only a single nucleotide difference between the wild-type and mutant constructs.

Additionally, were these results based on biological replicates or technical replicates within a single experiment?

**Response:** Thank you for this perceptive question. Importantly, our PB2-N460S and NP-I163T amino acid substitutions each result from a single nucleotide change in the coding sequence. Such a minimal change can nonetheless boost mRNA levels—likely by enhancing local RNA stability, for example through subtle remodeling of secondary structure that limits nuclease access or by creating/disrupting short motifs for RNA-binding proteins or microRNAs. Finally, we confirmed these effects across three independent biological replicates.

8. Clarification is needed on the mutations that were introduced into the PA and which PA variant was used throughout your experiments. For instance, in the section “Virus rescue by reverse genetics” (line 349-351), which amino acid substitutions were introduced into the PA. In line 352 and 398-399, please clarify whether the mutations refer only to NP and PB2, or to PA as well.

**Response:** Thank you for the suggestion. We have added the following sentence to the “Virus rescue by reverse genetics” section: “For clarity, GZ0215-01 naturally encodes PB2 residues 261M and 460N, PA residues 293N, 338K, and 495M, and NP residue 163I.”

9. Discussion (line 282-283): The sentence refers to a high prevalence of PB2-460S and NP-163T substitutions in IBV sequences retrieved from the GISAID database. However, the term “high” remains vague. Please provide exact proportions of sequences carrying these substitutions and indicate whether PB2-460S and NP-163T independently or in combination or both is meant in the statement. What about the spatial and temporal distribution of sequences with PB2-460S and NP-163T (have thy been consistently present in circulating strains over an extended time period or is there a recent increase).

**Response:** We have done prevalence analysis of PB2-460 and NP-163 across all available IBV genomes from GISAID (Figure α). This revealed that the PB2-460N and NP-163I substitutions occurs at <4% and <0.2% annually, respectively, whereas the PB2-460S/NP-163T combination is found in >99% of sequences. We have incorporated these data into the Discussion (lines 341–348) to highlight the near‐fixation of S/T in circulating IBVs. We did not undertake a geographic distribution analysis, since the overwhelming global dominance of PB2-460S/NP-163T precludes meaningful regional comparisons.

**Figure α. Annual prevalence of PB2-460 and NP-163 variants in global IBV genomes**

10. It appears that a previous publication reporting on Influenza B virus animal models [https://doi.org/10.1186/s12985-019-1171-3] used the same patient sample employed in the current study. To aid the reader’s understanding, I recommend citing the earlier study and briefly contextualize the results.

**Response:** Thank you for pointing this out. We have now cited the earlier animal‐model study that used the same clinical specimen and added a brief contextual in the Introduction (lines 107–111):

“In our previous study, we isolated the parental B/Guangzhou/0215/2012 (GZ0215) viral isolate from a patient in Guangzhou, China, and characterized it in ferret, tree shrew, and mouse models. That work found mild symptoms in ferrets and tree shrews but lethality in mice [34]”

11. Results (line 123–124): “we employed reverse genetics to generate four recombinant IBV viruses in the GZ0215-01 genetic background using reverse genetics”. The term “reverse genetics” is duplicated and should be removed.

**Response:** The duplicated “reverse genetics” has been removed.

12. Results (line 212–213): “It has been reported that the role of PB2-NP interactions in viral ribonucleoprotein (vRNP) assembly and RNA synthesis regulation [25, 26].” The statement of the sentence is unclear since essential parts are missing. Please rephrase for clarity.

**Response:** We have rephrased the sentence (lines 268–269) to: “Previous studies have demonstrated that interaction between PB2 and NP is critical for viral ribonucleoprotein (vRNP) assembly and regulation of viral RNA synthesis.”

13. Histopathology (line 433): Please verify the statement that “5 mm” tissue sections were mounted on glass slides – this appears unusually thick for histopathology.

**Response:** Thank you for pointing this out. We have corrected line 531 to state that 5 μm (not 5 mm) tissue sections were mounted on glass slides.

14. Table 1 (line 655): There appears to be a mistake at amino acid positions 460 in PB2. The codons AGT (Serine) and AAT (Asparagine) do not match the order of the indicated residues. Please verify and correct.

**Response:** This has been corrected in Table 1.

15. The caption of Figure 1 seems to be mislabeled and should be revised. Specifically, (line 667): “(C) Body weight changes and (D) survival rates” does not match the data shown. Additionally, line 669 – 671 “(E) The median lethal doses (LD50) of the two isolates were calculated based on the survival rates from panel D. Whole viral genome analysis via Sanger sequencing…” should be clarified.

**Response:** Figure 1 legend has been revised to correctly label body-weight changes (panel C) and survival rates (panel D), and to clarify that panel E shows LD_50_ calculations based on the survival data.

16. Caption of Figure 1 (line 672): “resulted from codon changes aat1401-1403agt and atc547-549acc, respectively” The nucleotide position stated here differs from what is shown in Figure 1 panel (E) and Table 1. Please verify.

**Response:** We have updated the codon positions in the Figure 1 legend to match those shown in panel E and Table 1.

17. Figure 4 (line 710): There is no panel (E) presented in the figure referenced. Please revise.

**Response:** Thank you for catching this. This has been correct in line 860.

18. Formatting: Please check the format of the p values and references. Reference 9 includes a partial “competing interest” statement and should be revised, while few references do not provide a digital object identifier (doi).

**Response:** Thank you for pointing this out. We have reformatted all p-values throughout the manuscript, removed the stray “competing interest” text from Reference 9, and added DOIs to all references that were missing them.
